# Genome-wide identification of the C2H2 zinc finger gene family in *Populus euphratica* and the functional analysis of *PeZFP38* under salt stress

**DOI:** 10.3389/fpls.2026.1754976

**Published:** 2026-01-26

**Authors:** Yazhi Zhao, Zhengquan He, Lijiao Fan, Huan Liu, Huiling Chen, Yuying Yang, Na Fan, Xiaojiao Han, Zhuchou Lu, Renying Zhuo

**Affiliations:** 1 Key Laboratory of Three Gorges Regional Plant Genetic & Germplasm Enhancement (CTGU), Biotechnology Research Center, China Three Gorges University, Yichang, China; 2 State Key Laboratory of Tree Genetic and Breeding, Research Institute of Subtropical Forestry, Chinese Academy of Forestry, Hangzhou, China; 3 Zhejiang Key Laboratory of Forest Genetics and Breeding, Research Institute of Subtropical Forestry, Chinese Academy of Forestry, Hangzhou, China

**Keywords:** C2H2-ZFP gene family, expression patterns, PeZFP38 gene, *Populus euphratica*, salt stress

## Abstract

The C2H2 zinc finger protein (C2H2-ZFP) is a large transcription factor (TF) in plants, widely distributed across plants and playing crucial roles in growth, development, and responses to abiotic stress. However, most studies on the C2H2-ZFP gene family have mainly focused on model plants. In this study, we systematically identified the C2H2-ZFP gene family members in *Populus euphratica*, a tree species with high tolerance to salt and alkali stress, by analyzing gene localizations, conserved motifs, gene structures, and phylogenetic relationships. A total of 67 members of the *P. euphratica* C2H2-ZFP gene family were identified and were divided into five subfamilies. Promoter analysis revealed numerous cis-acting elements related to development, hormones, and abiotic stress. Both tandem and segmental duplications were identified as the main driving forces behind the expansion of the PeZFP gene family. Expression profiling showed that most *PeZFPs* exhibit tissue-specific expression patterns and respond to salt stress. Among them, *PeZFP38* was strongly induced by salt stress in roots, stems, and leaves, with expression levels increased by 4.3–10.2-fold, 6–10.4-fold, and 28–63.7-fold, respectively. Subcellular localization demonstrated that PeZFP38 is a nuclear protein. Functional assays showed that transient overexpression of *PeZFP38* in poplar leaves enhanced salt tolerance, and stable overexpression of *PeZFP38* in *Arabidopsis thaliana* increased biomass (~68% fresh weight), enhanced antioxidant enzyme activities (e.g., SOD activity reached 1.7-fold that of WT), and reduced oxidative damage (~30% MDA decrease). These results suggest that *PeZFP38* may play a role in enhancing salt tolerance by integrating ABA signaling with ROS scavenging systems. Collectively, this study systematically deciphers the evolutionary relationships and expression patterns of the C2H2-ZFP family in *P. euphratica*. For the first time, it functionally identifies the positive regulatory role of *PeZFP38* in salt stress response. These findings provide novel genetic resources and a theoretical basis for understanding stress resistance mechanisms and genetic improvement in forest trees.

## 1 Introduction

Forests represent one of the largest terrestrial carbon reservoir, which is essential for mitigating global warming (Polle and Rennenberg, 2019). Abiotic stress is the primary factor limiting the growth of forest trees, with salt stress being particularly harmful. Salt stress impairs photosynthesis through osmotic stress and ionic toxicity, disrupts metabolic balance, and induces oxidative stress. These effects severely inhibit tree growth (Hao et al., 2021). Understanding the salt tolerance mechanism of forest trees is crucial for selecting stress-resistant species and utilizing them to restore saline soils, which is vital for ecological security (Hao et al., 2021).

To elucidate the mechanisms underlying salt tolerance in forest trees, it is essential to explore the molecular regulatory networks that govern their response to salt stress. Among the numerous molecular components involved, TFs serve as core regulators by activating or repressing downstream target genes to mediate plant abiotic stress responses (Admas et al., 2025; Bokolia et al., 2024). TFs such as NAC (Chen and Xia, 2025), WRKY, bHLH (Filiz and Kurt, 2021), MYB (Wang et al., 2021), and C2H2-ZFP (Han et al., 2020) function as molecular switches within signaling pathways (Liu et al., 2022). Among these, ZFPs constitute the largest family of transcriptional regulators in plants, characterized by finger-like domains containing two cysteine and two histidine residues (Zhou et al., 2022). The ZFP domain is highly conserved and features a common sequence motif, CX_2-4_CX_3_FX_5_LX_2_HX_3-5_H, comprising approximately 20–30 amino acids that fold into a spatial structure consisting of two β-sheets and one α-helix (Zhou et al., 2022). ZFPs are classified into types such as C2H2, C2HC, C2HC5, C3HC4, CCCH, C4, C4HC3, C6, and C8 based on the number and arrangement of cysteine and histidine residues that coordinate zinc ions (Han et al., 2020; Shao et al., 2023). In plants, the zinc finger domain of C2H2-ZFPs typically contains a highly conserved QALGGH motif, which may play a role in regulating plant-specific biological processes (Pu et al., 2021).

The C2H2-ZFP gene family is widespread across the plant kingdom and involved in a wide range of biological functions. These encompass key aspects of plant growth, development, and adaptive reactions to environmental challenges like drought, high salinity, and cold (Nikraftar et al., 2024). In model plants like *Arabidopsis thaliana* (176) (Lu et al., 2024), tomato (*Solanum lycopersicum*, 99) (Zhao et al., 2020b), and poplar (*Populus trichocarpa*, 109) (Liu et al., 2015), the family size, evolutionary relationships, and functions of several members have been well characterized. For example, the first plant C2H2 gene, *TAZ*, identified in petunias, is involved in petal development, while *ZFP5* in *A. thaliana* regulates ethylene signaling and root hair development (Zhang et al., 2022a). In *Arabidopsis*, *AtSIZ1* enhances salt tolerance through the regulation of ionic homeostasis and osmotic balance (Han et al., 2019). Studies in apple (*Malus domestica*) have revealed that *ZAT5* acts as a negative regulator of salt tolerance by enhancing plant sensitivity to salinity stress (Wang et al., 2022a). In *P. euphratica*, *PeSTZ1* enhances cold tolerance by regulating antioxidant enzyme gene expression (He et al., 2019). In rice, overexpression of *OsZFP15* promotes ROS production and compromises cellular oxidative stress tolerance, yet simultaneously improves plant performance under salinity and drought conditions (Wang et al., 2022b). Conversely, in banana, overexpression of *MaC2H2-2* and *MaC2H2-3* leads to a suppression of the signaling pathway associated with cold stress (Han and Fu, 2019).

As a keystone species of desert riparian forests, *P. euphratica* possesses exceptional salt and drought tolerance, serving as an ideal system for studying stress resistance mechanisms in trees (Jia et al., 2020). *P. euphratica* can survive under extreme environmental conditions through mechanisms such as ion homeostasis, reactive oxygen species (ROS) scavenging, and cell wall modification (Niu et al., 2024). Comparative genomic studies have revealed significant expansion of stress-related gene families in its genome (Jia et al., 2020), enhancing ion regulation (e.g., sodium exclusion and potassium accumulation) and maintaining water balance under stress conditions (Zhao et al., 2020a). These results suggest adaptive evolution of its transcriptional regulatory networks.

Current studies on C2H2-ZFPs have primarily focused on model species such as *A. thaliana* and rice. However, systematic identification, expression profiling, and functional validation of this family remain lacking in *P. euphratica*. In this study, we systematically identified the evolutionary characteristics and expression patterns of the C2H2-ZFP gene family in *P. euphratica* based on the whole-genome sequence. Furthermore, we focused on a candidate gene, *PeZFP38*, whose promoter is enriched with stress-responsive cis-acting elements. *PeZFP38* belongs to one subfamily, which contains *P. euphratica*-specific clades, and is rapidly and persistently induced in roots, stems, and leaves under salt stress. Heterologous overexpression assays confirmed that *PeZFP38* significantly enhances salt tolerance in both 84K poplar and *Arabidopsis*. These findings provide a framework for elucidating the molecular basis of adaptation to environmental stress in *P. euphratica* and offer valuable genetic resources for forest tree improvement (Ndayambaza et al., 2025).

## 2 Materials and methods

### 2.1 Plant materials and growth conditions

The plant materials used in this experiment were all obtained from and preserved in the Laboratory of Forest Genetics and Breeding, Subtropical Forestry Research Institute, Chinese Academy of Forestry (Hangzhou, Zhejiang). *P. euphratica* (desert poplar) was selected as a natural seedling clonal line. Young shoots of *P. euphratica* were collected from a forest in Jiuquan, Gansu Province. Sterilized stem segments were cultured on rooting medium to generate aseptic seedlings, which were used for subsequent experiments. 84K poplar (*Populus alba* × *Populus glandulosa*) was a hybrid first-generation clonal line provided by the Chinese Academy of Forestry. Both *P. euphratica* and 84K poplar were cultured aseptically *in vitro* under constant conditions of 24°C, 16 hours (h) light/8 h dark photoperiod, and 50%–60% relative humidity. The wild-type (WT) *A. thaliana* used was the Columbia ecotype (Col-0). *A. thaliana* seeds were stored at -4°C and subsequently germinated on half-strength Murashige and Skoog (MS) solid medium (M519, PhytoTech Labs, US) before cultivation. The materials used in this experiment comprised 3-month-old aseptic *P. euphratica* seedlings, 1-month-old aseptic 84K poplar seedlings, and 2-week-old/1-month-old *A. thaliana* seedlings. All materials were in consistent growth condition and free from disease.

### 2.2 Methods

#### 2.2.1 Identification of C2H2-ZFP gene family in *P. euphratica*

*P. euphratica* genome and protein sequences were downloaded from the China National Center for Bioinformation (https://ngdc.cncb.ac.cn/omix/). Genomic data for *A. thaliana* and *P. trichocarpa* were obtained from the Ensembl Plants database (https://plants.ensembl.org/index.html). To comprehensively identify all C2H2-ZFP gene family members in *P. euphratica*, a dual strategy was employed. First, the hidden Markov model (HMM) profile corresponding to the C2H2-ZFP domain (PF00096) was acquired from the Pfam database (http://www.ebi.ac.uk/interpro/). This profile was used as a query to search for the *C2H2-ZFP* genes in the *P. euphratica* genome using TBtools-II software (version 2.371) (Chen et al., 2020). Second, 173 known C2H2-ZFP proteins from *A. thaliana* were used as query sequences for BLASTP searches against the *P. euphratica* protein database with an E-value < e^-5^. The results from both searches were merged, and redundant sequences were removed. All candidate proteins were then validated for the presence of C2H2-ZFP domains using SMART and the NCBI Batch CD-Search tool.

#### 2.2.2 Chromosomal location and characterization analysis

The 67 identified *PeZFP* genes were renamed according to their physical positions on the chromosomes of *P. euphratica*. The Protein Parameter Calculator function in TBtools-II software was used to determine the number of amino acids, molecular weight, isoelectric point, instability index, aliphatic index, and grand average of hydropathicity for each PeZFP protein. The subcellular localization of the identified proteins was predicted using the WoLF PSORT (https://wolfpsort.hgc.jp) online tool.

#### 2.2.3 Phylogenetic analysis

An unrooted phylogenetic tree was generated using 67 full-length PeZFP proteins from *P. euphratica* and 173 full-length AtZFP proteins from *A. thaliana*. The maximum likelihood (ML) method, implemented in MEGA-X, was used with 1000 bootstrap replicates. The resulting phylogenetic tree was further classified and visualized using the iTOL online platform (https://itol.embl.de/).

#### 2.2.4 Conserved motif and gene structure analysis

The conserved motifs of the PeZFP proteins were predicted utilizing the MEME program (version 5.5.8) (https://meme-suite.org/meme/), with the maximum number of motifs constrained to ten. Exon-intron structures of *PeZFP* genes were derived from the genome annotation file of *P. euphratica.* The phylogenetic tree, conserved motifs, and gene structures of *PeZFPs* were subsequently integrated and visualized using TBtools-II software (Chen et al., 2020).

#### 2.2.5 Promoter cis*-*acting elements analysis

The 2-kb upstream promoter sequence of each *PeZFP* gene, relative to the translation start site, was extracted using TBtools-II software. The sequences were analyzed using the PlantCARE database (https://bioinformatics.psb.ugent.be/webtools/plantcare/html/) to identify cis-acting regulatory elements (CREs). The types and frequencies of CREs were summarized and visualized using Microsoft Excel and the Gene Structure Viewer tool in TBtools-II.

#### 2.2.6 Collinearity and gene duplication event analysis

The MCScanX module in TBtools-II was used to analyze collinearity relationships within the PeZFP gene family. The results were visualized using the Advanced Circos tool in TBtools-II. Interspecific collinearity between *P. euphratica* and *A. thaliana* or *P. trichocarpa* was visualized using the Dual Synteny Plot tool in TBtools-II, respectively. The ratio of non-synonymous (Ka) to synonymous (Ks) nucleotide substitutions for duplicated gene pairs was calculated using the Simple Ka/Ks Calculator to determine selection patterns.

#### 2.2.7 Expression pattern analysis under salt stress

*P. euphratica* seedlings were hydroponically cultured in 1/2 Hoagland nutrient solution (NSP1020, Coolaber, China) for 3 days (d) before salt treatment. Salt treatment was performed by adding 300 mM NaCl (Ge et al., 2022) to the nutrient solution. All samples of roots, stems, and leaves were harvested at 0 h, 6 h, 12 h, 1 d, 4 d, and 7 d (Li et al., 2024) after salt treatment for transcriptome sequencing. RNA-seq libraries were constructed and sequenced on an Illumina platform by Genedenovo Biotechnology Co., Ltd (Guangzhou, China). Clean reads were aligned to the *P. euphratica* v2.0 genome and quantified following standard pipelines (Li and Dewey, 2011; Wang et al., 2009). Raw data are available in the China National Center for Bioinformation database under accession PRJCA050032. Expression levels were measured in TPM value. Expression levels of *PeZFPs* across time points and tissues were organized and quantified in Microsoft Excel. Data with no detectable expression were excluded from statistical analysis. Three biological replicates were used. Data were normalized using Z-score standardization by column or by row before clustering. Heatmaps were generated using TBtools for visualization.

#### 2.2.8 Subcellular localization

The CDS sequence of the *PeZFP38* (with the stop codon removed) was cloned into the pBI121-GFP vector via *Xba*I and *Kpn*I sites to construct the fusion expression vector pBI121-*PeZFP38*-GFP. The primers used are listed in **Supplementary Table S5**. Using *P. euphratica* cDNA as the template, the product was amplified with Phanta Max Master Mix (P525-03, Vazyme, China). After gel purification, ligation, and transformation, positive clones were verified by sequencing (Shangya Bio, Hangzhou, China). The recombinant construct was transformed into the *Agrobacterium* strain GV3101 (CC96304, TOLOBIO Biotech, China). Positive *Agrobacterium* cultures were grown in LB medium containing the appropriate antibiotics until OD_600_ reached 0.8. Cells were collected by centrifugation and resuspended in infiltration buffer (containing 10 mM MES, 20 mM MgCl_2_, and 200 μM acetosyringone) to an OD_600_ of 1.0, and incubated at room temperature for 2 h. For infiltration, bacterial suspensions containing the target or control vector were mixed at a 1:1 ratio with the nuclear marker p2300-35S-D53-mCherry. The mixed suspension was then infiltrated into the abaxial side of *Nicotiana benthamiana* leaves. After infiltration, the plants were kept in the dark for 12 h to reduce fluorescent protein photodegradation, then transferred to normal light conditions for 36 h to promote protein folding and accumulation. The subcellular localization of the fusion protein was examined using a confocal laser scanning microscope (LSM900, Zeiss, Germany).

#### 2.2.9 Transient *PeZFP38* gene expression analysis in poplar leaves

Healthy one-month-old sterile seedlings of 84K poplar with uniform growth were used in the transient expression experiment. Leaf discs (6 mm in diameter) collected from the 2^nd^ to 4^th^ leaves at the morphological apex. These discs were then transiently transformed via *Agrobacterium* GV3101 carrying either the experimental plasmid pBI121-*PeZFP38*-GFP or the control plasmid pBI121-GFP, as referenced in (Chen et al., 2025; Lin et al., 2017). The transformed leaves were first incubated on 1/4 MS solid medium under dark conditions for 2 d, and then transferred to 1/4 MS liquid medium containing 0, 100, 300, or 500 mM NaCl for 7 d. Based on previous studies (Chen et al., 2025; Lei et al., 2024; Li et al., 2024), the NaCl concentration gradient was designed to cover a range from mild to extreme salt stress. Chlorophyll content was measured using a microplate reader (SpectraMax iD5, MD, USA) as previously described (Tyagi et al., 2023) to evaluate the effect of salt treatment. All experiments included at least three biological replicates.

#### 2.2.10 Overexpression of *PeZFP38* in *A. thaliana*

The recombinant plasmid pBI121-*PeZFP38*-GFP was introduced into *A. thaliana* using the floral dip method (Ali et al., 2024) with *Agrobacterium* strain GV3101. Three independent T1 transgenic lines confirmed by genomic PCR and identified as high-expression lines by Quantitative Real-Time PCR (qRT-PCR) were selected and propagated to obtain homozygous T3 plants for all subsequent experiments. For the salt stress plate experiments, 7d seedlings grown on 1/2 MS medium were transferred to medium supplemented with 0, 50, 100, or 150 mM NaCl (Ma et al., 2023; Qu et al., 2023). After 7 d of growth, biomass and root length were measured. For the hydroponic salt stress assay, 20d seedlings cultured in 1/2 Hoagland nutrient solution were treated with 100 mM NaCl for 7 d (Qu et al., 2023), after which growth parameters and antioxidant enzyme activities were analyzed (Deng et al., 2024). All experiments included at least three biological replicates. Histochemical staining with nitroblue tetrazolium chloride (NBT) and 3,3′−diaminobenzidine (DAB) was performed to detect the accumulation of ROS in leaves (He et al., 2020).

### 2.3 Statistical analysis

All data were statistically analyzed using SPSS (version 27.0) software and visualized using GraphPad Prism (version 9.5.0) software. Data represent the mean ± standard deviation (SD) from at least three independent biological replicates. Before conducting formal statistical tests, the normality of data distribution and homogeneity of variances were verified to satisfy the assumptions for parametric tests. Difference between two groups was assessed by two-tailed unpaired Student**’**s t-test with *p* < 0.05. The significance of differences between multiple groups was evaluated by one-way ANOVA followed by LSD test (*p* < 0.05).

## 3 Results

### 3.1 Identification of C2H2-ZFP gene family members in *P. euphratica*

**Figure 1:**
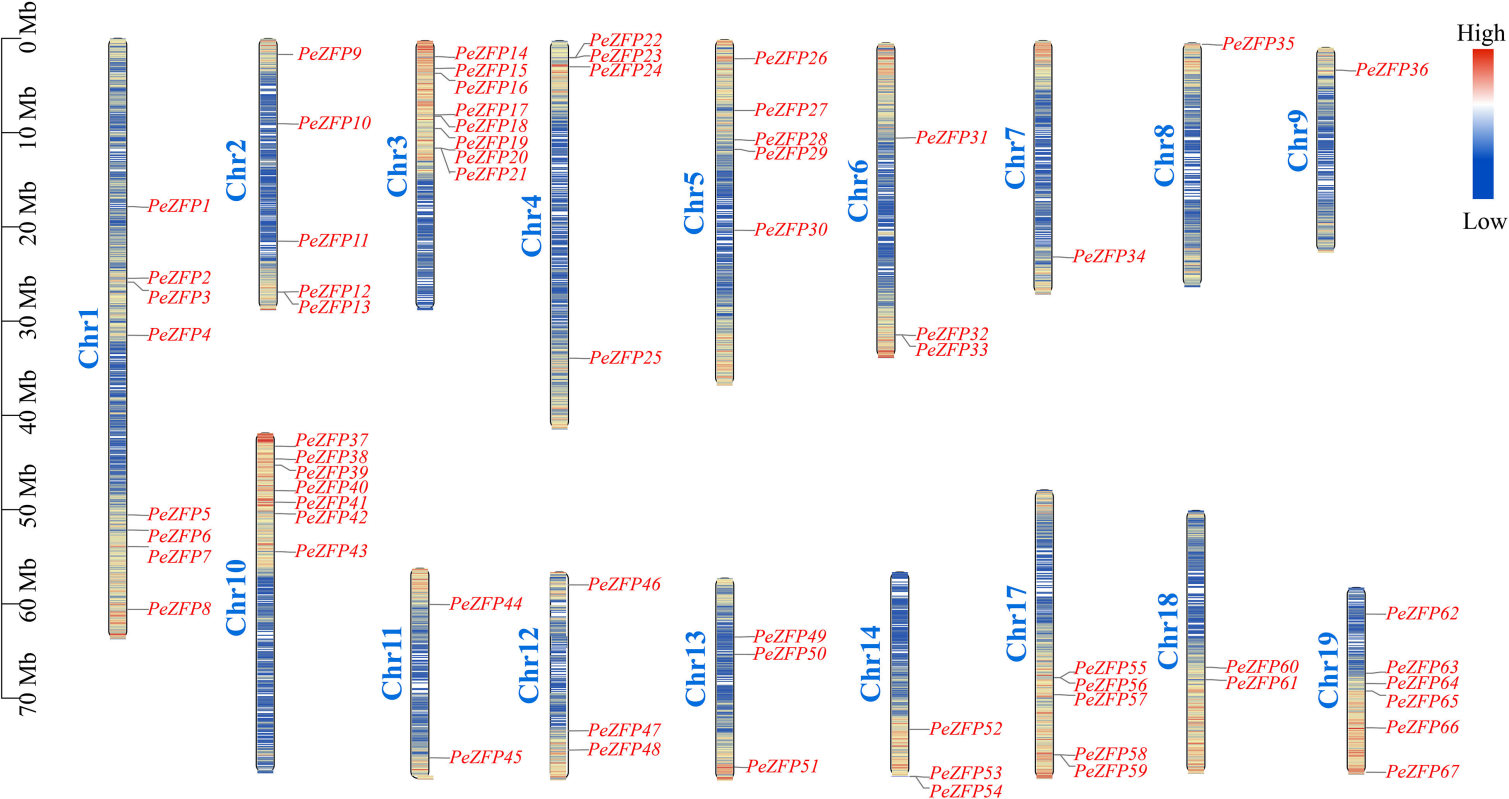
Chromosomal distribution of *PeZFPs* in *P. euphratica*. The lengths of the 17 chromosomes are represented on the left in megabases (Mb). Black lines indicate the precise locus of each gene on its corresponding chromosome, while the color gradient along the chromosomes represents gene density.

Using BLASTP and HMM searches for the C2H2-ZFP domain in the *P. euphratica* genome database, a total of 67 *C2H2-ZFP* genes were identified. These genes were sequentially renamed *PeZFP1* to *PeZFP67* based on their chromosomal locations. These genes were distributed across chromosomes 1 to 19 of *P. euphratica*, with no genes located on chromosomes 15 and 16. Chromosomes 1 and 3 contained the highest numbers of *PeZFPs*, while only one gene was detected on chromosomes 7, 8, and 9 (**Figure 1**).

Analysis of the physicochemical characteristics of the PeZFP proteins is summarized in **Supplementary Table S1**.The amino acids lengths ranged from 126 to 593, averaging 310. Corresponding molecular weights ranged from 14.67 kDa to 67.96 kDa, with an average of 34.17 kDa. The theoretical isoelectric point showed a spectrum from 4.87 to 9.76, averaging 7.73. The instability index varied between 39.27 and 74.29, averaging 57.08. Unlike PeZFP20, most PeZFPs were predicted to be unstable. The aliphatic index ranged from 40.59 to 86.49, with an average of 60.57. The grand average of hydropathicity varied from −1.202 to −0.134, averaging of −0.72. Subcellular localization predictions indicated that all PeZFPs may localize to the nucleus, which is consistent with the typical characteristics of C2H2-ZFP TFs (Huang et al., 2024).

### 3.2 Phylogenetic analysis of PeZFPs in *P. euphratica*

**Figure 2:**
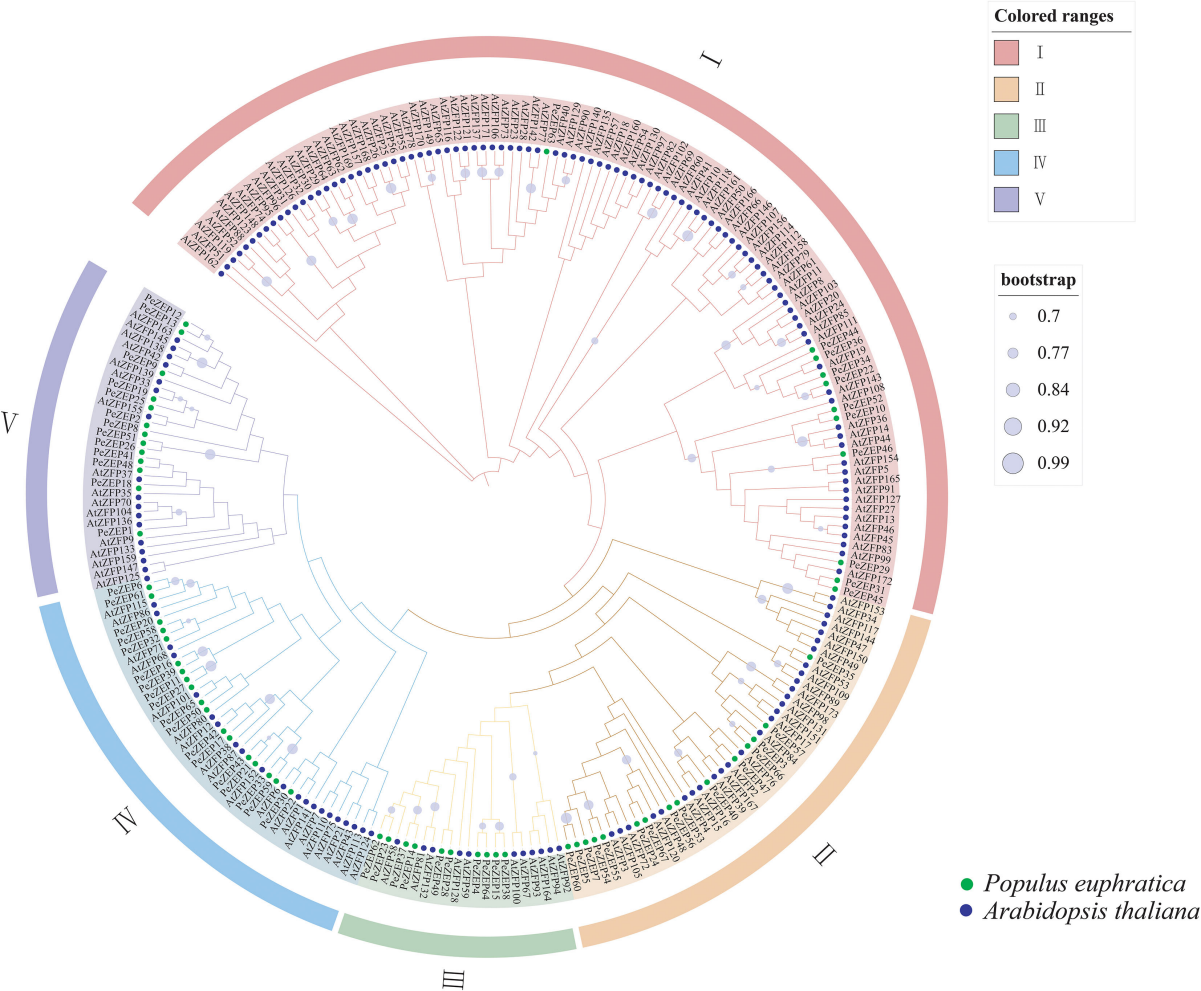
Phylogenetic tree of the PeZFP gene family in *P. euphratica* and *A. thaliana*. The unrooted tree was constructed using the ML method based on PeZFP and AtZFP protein sequences. The tree is divided into five subfamilies, each highlighted with a distinct color. Branches are annotated with ML bootstrap values (≥70%). AtZFPs are denoted by solid purple circles, whereas PeZFPs are indicated by solid green circles.

To elucidate the evolutionary relationships and functional diversification of PeZFPs, an unrooted phylogenetic tree was constructed using the maximum likelihood (ML) method based on 67 PeZFP protein sequences and 173 AtZFP protein sequences. AtZFP proteins were named based on their chromosomal locations in *A. thaliana*. Based on their branch clustering patterns, protein sequence similarities, and previously established classifications (Arrey-Salas et al., 2021; Zhou et al., 2024), the PeZFPs were classified into five subfamilies (I, II, III, IV, V), containing 11, 15, 10, 18, and 13 members, respectively (**Figure 2**). Key branching nodes received strong bootstrap support (≥70%, represented by color gradients), confirming the reliability of the classification.

Phylogenetic analysis revealed that PeZFPs and AtZFPs frequently formed cross−species mixed branches within each subfamily. This pattern was particularly evident in subfamily I, where the two groups were closely nested, indicating that these subfamilies likely originated prior to species divergence and may have retained conserved functions. Notably, subfamilies II–V contained several clades consisting exclusively of *P. euphratica* members, suggesting lineage−specific expansion or functional specialization during adaptation to arid and saline environments.

### 3.3 Conserved motif and gene structure analysis

**Figure 3:**
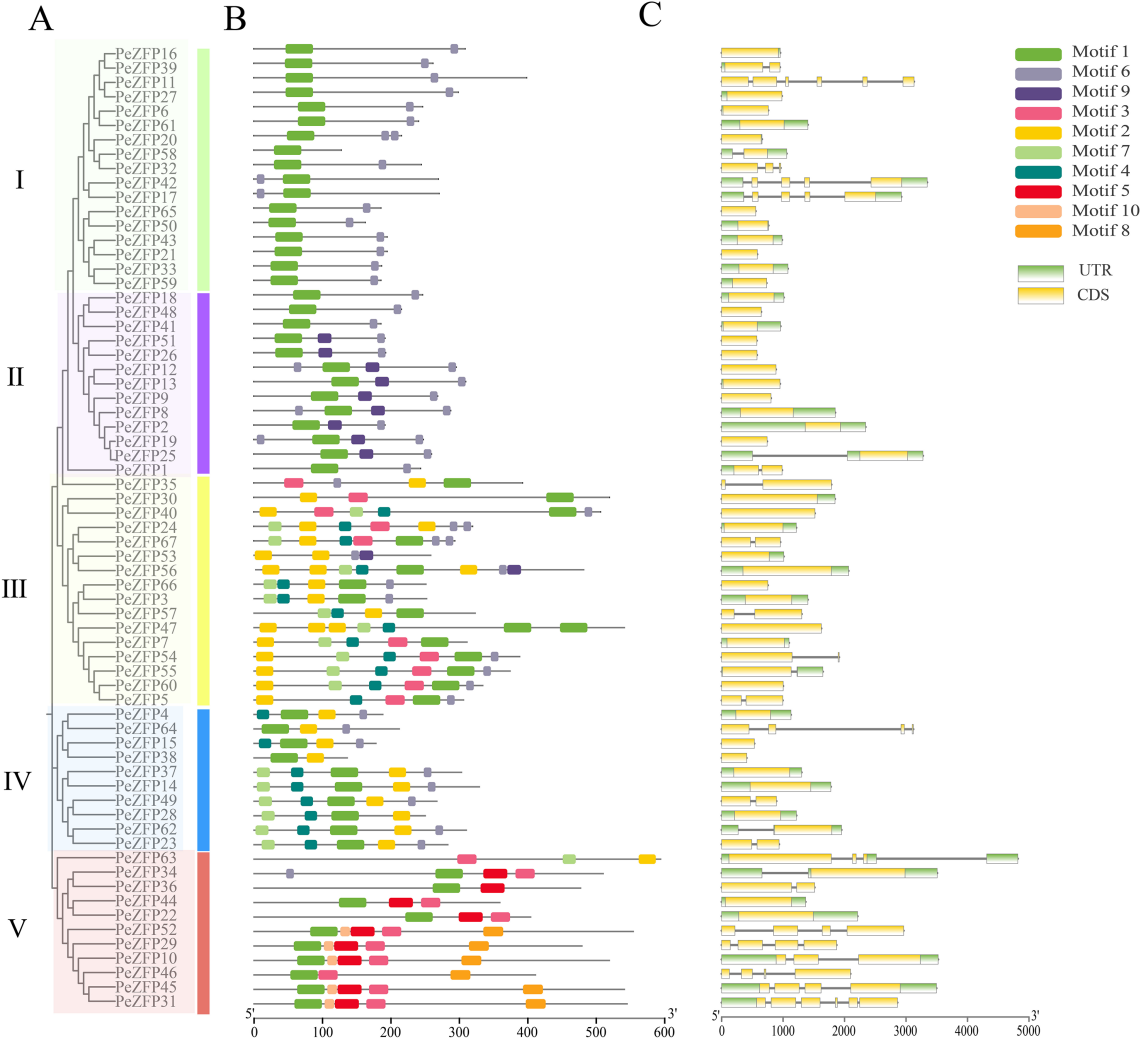
Conserved motifs and gene structure of PeZFPs in *P. euphratica*. **(A)** Phylogenetic tree of PeZFPs, with distinct subfamilies represented by differently colored blocks. **(B)** Conserved motif compositions of PeZFPs, ten identified motifs are illustrated by distinct color-coded boxes. **(C)** Gene structure organization of *PeZFPs*, where exons are shown in yellow, untranslated regions in green, and introns as black straight lines. The scale bar represents gene length in kilobases (Kb).

Phylogenetic analysis based on all PeZFP protein sequences revealed that these proteins can be classified into five subfamilies (I–V) according to evolutionary relationships and sequence similarity (**Figure 3A**). These subfamilies correspond to subfamilies IV, V, II, III, and I in the phylogenetic tree shown in **Figure 2**. To further elucidate the potential functions of PeZFPs, conserved motifs and gene structures were analyzed. Ten conserved motifs were identified among 67 PeZFP proteins (**Figure 3B**).

The number and arrangement of motifs were generally consistent within each subfamily, indicating functional conservation among members of the same subfamily. Except for PeZFP24, PeZFP53, and PeZFP63, all members contained motif 1, indicating that motif 1 was a highly conserved and potentially essential core element of the PeZFP gene family in *P. euphratica*. Notably, motif 1 and 2 contained the C2H2-ZFP sequences and the plant-specific motif QALGGH. Motif analysis revealed clear classification patterns, based on characteristic motifs, family members could be categorized into Q-type and M-type (**Supplementary Table S2**). Specifically, a total of 54 members (80.6%) contained the QALGGH motif and were classified as Q-type C2H2-ZFPs. The distribution of the Q-type showed subfamily specificity. All members of subfamilies I and II (excluding PeZFP25) contained one such motif, most members of subfamilies III and IV contained one or two such motif. The majority of members in subfamilies I–IV (e.g., those containing motif 6) possessed the characteristic EAR motif (LxLxL or DLNxxP), which typically conferred transcriptional repression activity (Wang et al., 2020). In contrast, members of subfamily V, which completely lacked this motif, were classified as M-type C2H2-ZFPs and generally did not contain an EAR motif. These results indicate that despite clear evolutionary divergence among the five subfamilies, protein sequences and potential function remain relatively conserved within each subfamily.

Gene structure analysis showed that 59.7% of the genes lack introns, while the rest contain 1 to 5 introns of varying lengths (**Figure 3C**). Furthermore, the absence of UTRs in 28 genes may stem from the incomplete nature of genome annotation, a limitation commonly encountered in genome-based studies that typically does not compromise coding sequence analysis. Exon–intron patterns were similar within subfamilies. For example, most members of subfamily II contained no introns and exhibited the simplest structure. In contrast, members of subfamily V, which lack the QALGGH motif, mostly contained multiple introns and displayed more complex structures. Such structural differences likely reflect distinct evolutionary histories involving gene duplication and intron gain/loss events, leading to unique structural features and potential regulatory complexity in each subfamily.

### 3.4 Promoter cis*-*acting elements analysis

**Figure 4:**
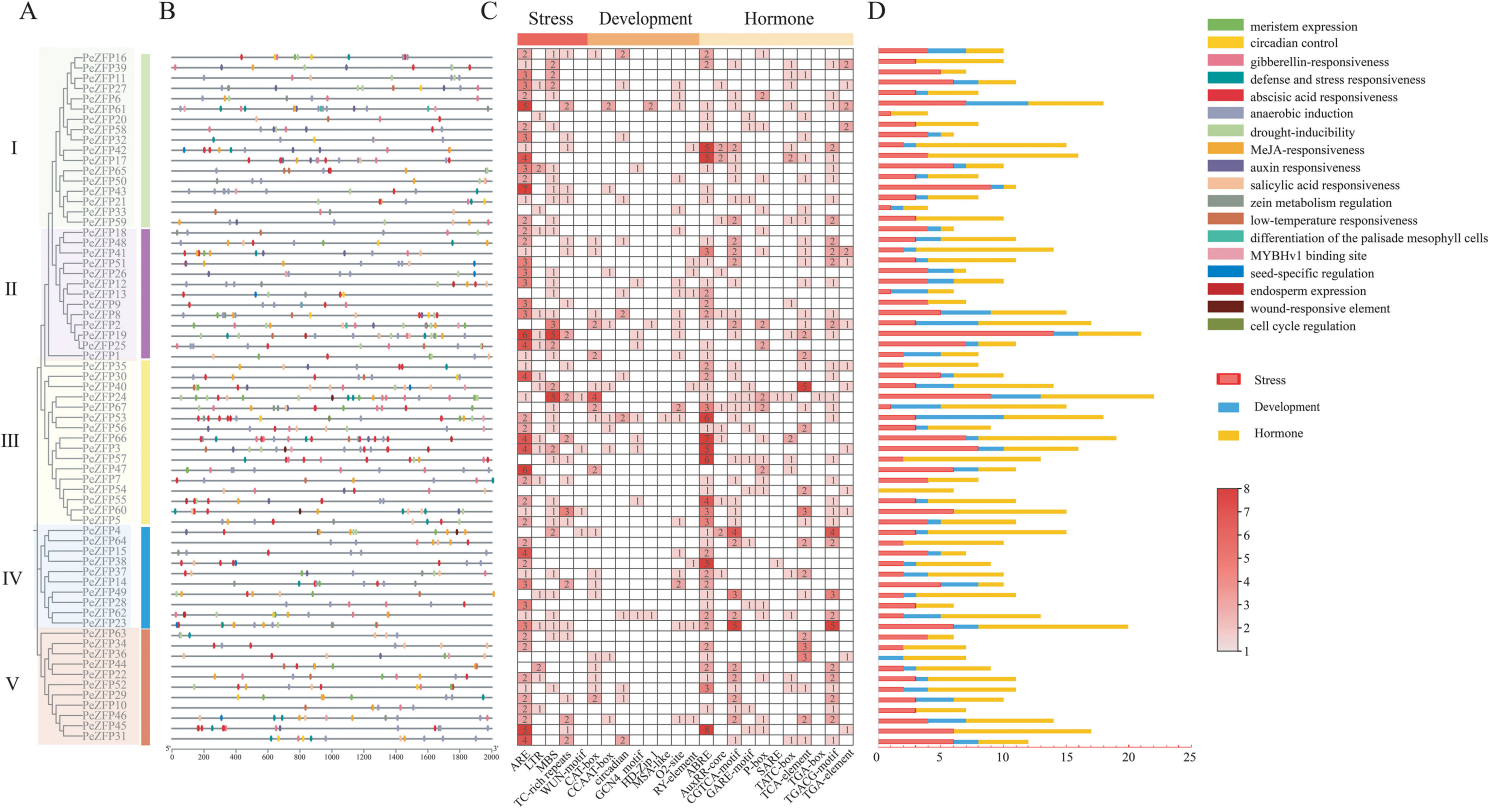
Distribution and number of CREs in *PeZFP* promoters of *P. euphratica*. **(A)** Phylogenetic tree of PeZFPs, with distinct subfamilies represented by differently colored blocks. **(B)** Distribution of CREs in *PeZFP* promoters. Different colored boxes represent different types of elements. **(C)** Number of CREs in *PeZFP* promoters. Darker red indicates a higher abundance of elements. **(D)** The histogram depicts the abundance of CREs across three major functional categories. The bars correspond to stress response (red), development (blue), and hormone response (yellow).

To further elucidate the potential regulatory mechanisms of *PeZFPs* in plant biological processes, the 2000 bp upstream promoter regions of all *PeZFPs* were analyzed. The results were arranged according to the phylogenetic tree (**Figure 4A**). A total of 18 types of CREs were identified, which were categorized into three major groups, such as stress response, plant development, and phytohormonal response. The distribution of CREs in the promoter regions exhibited a non-random clustering pattern, where specific types of elements tended to aggregate together, potentially forming synergistic transcriptional regulatory modules (**Figure 4B**).

Analysis revealed significant differences in the composition and abundance of CREs among different evolutionary subfamilies (**Figures. 4C, D**). This pattern was consistent with their phylogenetic relationships, characteristic motifs, and gene structural complexity. Specifically, subfamily II showed a relatively high proportion of development-related elements (e.g., metabolism expression and circadian control element), implying that its members may be primarily involved in the basal regulation of plant growth and development. In contrast, subfamilies III and IV were significantly enriched in stress- and hormone-responsive elements, with the anaerobic induction element ARE and the ABA-responsive element ABRE being the most prominent. This suggests that these two subfamilies may play core roles in mediating abiotic stress signal transduction (e.g., drought and hypoxia) and hormone-dependent stress responses. Subfamilies II (with simple gene structures, mostly Q-type) and V (with complex gene structures, mostly M-type) had more diverse and balanced CRE compositions, encompassing all three categories of elements. This indicates that their members may possess broader regulatory plasticity in biological functions.

Except for *PeZFP36* and *PeZFP54*, all *PeZFP* promoters contained stress-related elements, including those involved in responses to cold, drought, wounding, and hypoxia. Results suggest that these CREs may play potential roles in the adaptation of *P. euphratica* to abiotic stresses. Among all CREs, ARE, ABRE, CGTCA-motif, and MBS were the most abundant types, which are associated with hypoxia, ABA, methyl jasmonate, and drought responses, respectively (Biłas et al., 2016). These findings highlight these signaling pathways as the core components of the *PeZFPs* regulatory network in *P. euphratica*. For instance, *PeZFP19*, which contains multiple ARE, LTR, and MBS elements, and *PeZFP24*, which has the largest number of total CREs, are proposed as key candidate regulators of stress responses. Additionally, several elements were found to cooperate in the same regulatory pathways. For example, AuxRR-core, TGA-box, and TGA-element are associated with auxin responses, while P-box, TATC-box, and GARE-motif are all implicated in the regulation of gibberellin responses. This implies that *PeZFPs* may enhance the sensitivity and amplitude of plant responses to environmental signals through element synergy, thereby fine-tuning hormone demands and stress responses across different growth and developmental stages. Collectively, these complex variations in promoter architectures not only reveal the regulatory basis underlying the functional diversification of *PeZFPs* but also provide critical clues for interpreting their differential expression patterns in various tissues or under distinct stress conditions.

### 3.5 Collinearity and gene duplication event analysis

**Figure 5:**
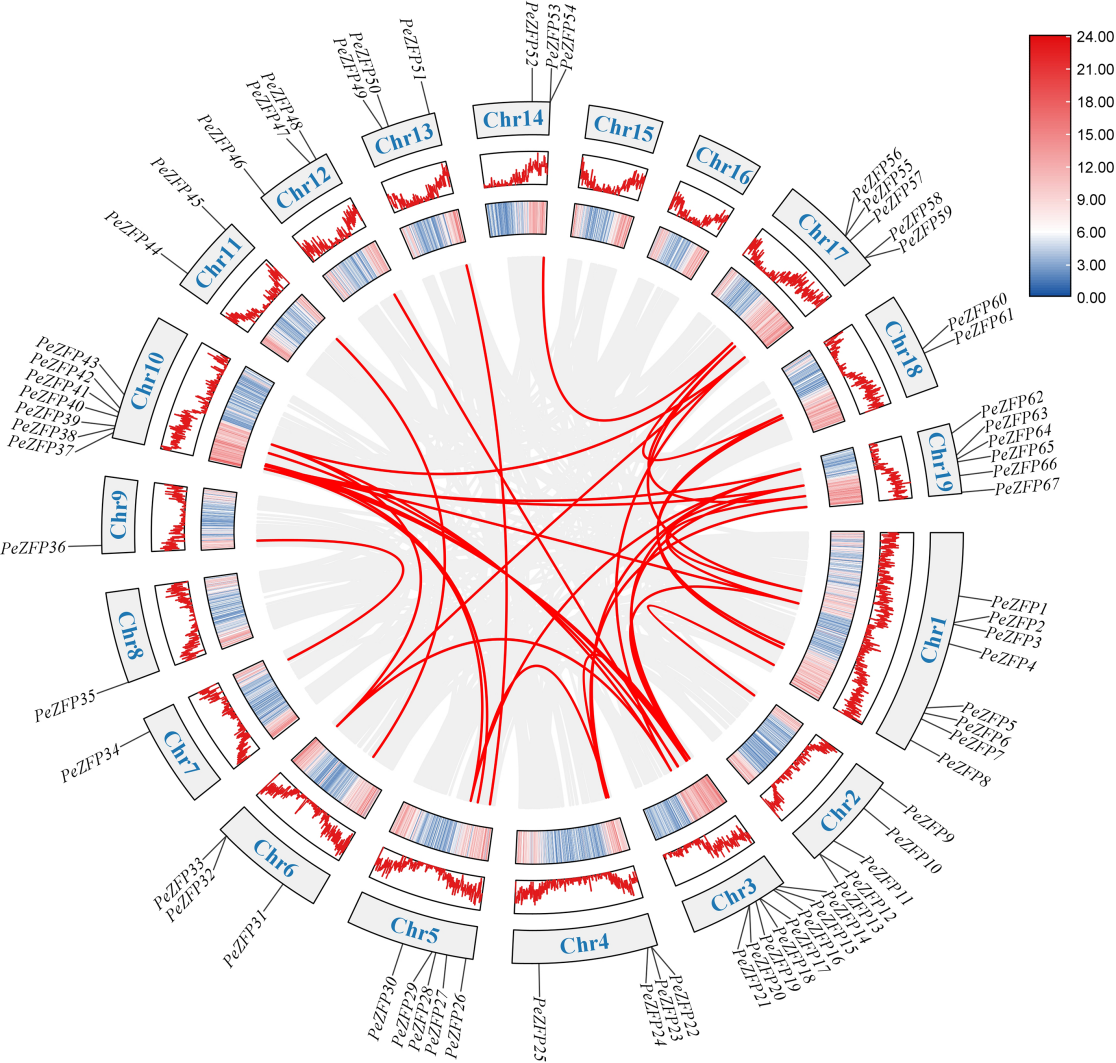
Intraspecific collinearity analysis of *PeZFP* genes in *P. euphratica*. The gray lines in the background denote collinearity pairs within the species, whereas the red lines signify the *PeZFP* gene pairs derived from segmental duplication events. The outermost circle displays the chromosome number, and the line map and heat map illustrate gene distribution and density along the chromosomes.

To elucidate the expansion mechanism and evolutionary drivers of the PeZFP gene family, we performed an integrated analysis of duplication events, selection pressure, and interspecific synteny. Tandem duplication and segmental duplication are two primary mechanisms driving gene family expansion (Huang et al., 2022). A total of 38 segmental duplication events and 8 pairs of tandem duplication events were identified (**Figure 5**), indicating that both types of duplication contributed to its expansion. Notably, tandemly duplicated genes (e.g., *PeZFP12*/*13*, *PeZFP32*/*33*) often clustered within the same phylogenetic clade or adjacent genomic regions, potentially facilitating the rapid formation of local gene clusters and functional specialization within subfamilies.

Analysis of Ka/Ks ratios for duplicated gene pairs (**Supplementary Table S3**) revealed that most values were significantly less than 1 (ranging from 0.12 to 0.62). Results indicated that this family has been under strong purifying selection (Alam et al., 2022) during evolution to maintain the zinc-finger structure and functional constraints. However, some gene pairs (e.g., *PeZFP14*/*23*) showed relatively higher Ka/Ks values, suggesting they may have undergone functional divergence. This observation aligns with their distribution in distinct phylogenetic subclades and differences in CRE composition.

**Figure 6:**
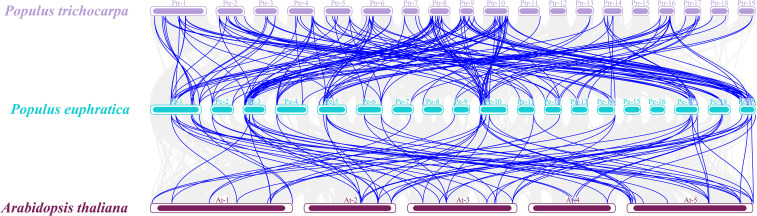
Collinearity analysis of *ZFPs* in *P. euphratica*, *P. trichocarpa* and *A. thaliana*. Purple, cyan, and brown represent the chromosomes of *P. trichocarpa*, *P. euphratica*, and *A. thaliana*, respectively. Gray lines in the background indicate all collinear gene pairs among the three species, whereas blue lines connecting distinct chromosomes highlight collinear *ZFP* gene pairs between *P. euphratica* and the other two species.

Interspecific synteny analysis further revealed the evolutionary conservation and specificity of *PeZFPs*. A total of 173 collinear gene pairs were identified between *P. euphratica* and *P. trichocarpa*, far more than the 81 pairs identified between *P. euphratica* and *A. thaliana* (**Figure 6**). These results suggest that a closer phylogenetic relationship between the two *Populus* species. Most conserved syntenic genes (e.g., *PeZFP16*, *PeZFP27*), which maintained orthologous relationships with both *P. trichocarpa* and *Arabidopsis*, contained typical QALGGH and EAR motifs, possessed simple gene structures, and were widely distributed across subfamilies I–IV. Their sequences and functions are likely stable and may be involved in fundamental transcriptional regulation. In contrast, *Populus*-specific retained gene pairs (e.g., *PeZFP40*, *PeZFP41* and their homologs) were found only within *Populus* and absent in synteny with *Arabidopsis*. These genes may have undergone functional differentiation, providing a potential genetic basis for the adaptation of *Populus* species, such as *P. euphratica*, to specific habitats. Together, these results demonstrate that the evolution and functional diversification of the PeZFP gene family were shaped by the combined effects of duplication events, purifying selection, and species-specific adaptation.

### 3.6 The expression patterns of *PeZFPs* in various tissues and under salt stress

**Figure 7:**
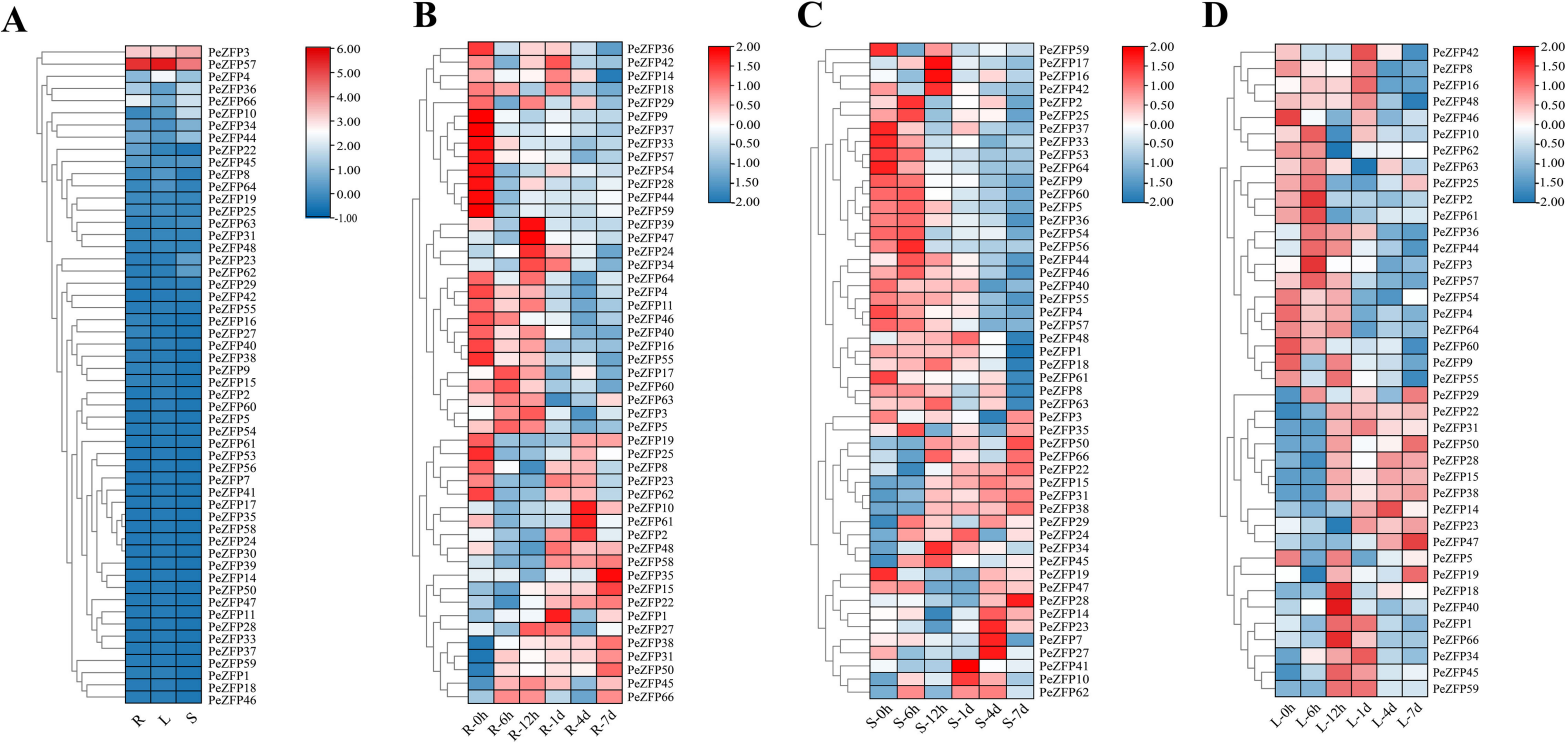
Expression profiles of *PeZFPs* in various tissues of *P. euphratica* under salt stress. **(A)** Tissue-specific expression patterns of *PeZFPs* under normal condition. **(B–D)** Dynamic expression patterns of *PeZFPs* in roots (R), stems (S), and leaves (L) under 300 mM NaCl stress.

Based on the analysis of promoter CREs, *PeZFPs* contained numerous stress-response elements. To further investigate their expression patterns and potential association with CREs, the expression of 67 *PeZFPs* in roots, stems, and leaves using transcriptome data of *P. euphratica* under salt treatment were analyzed. Heatmap were generated using TBtools to visualize the expression patterns (**Figure 7**).

Expression profiling revealed significant tissue-specific expression and differential responses to salt stress among *PeZFPs*. Under normal conditions, most genes were expressed in roots, stems, and leaves of *P. euphratica*, indicating their functional diversity (**Figure 7A**). Some *PeZFPs* showed no detectable expression. Specifically, four members (*PeZFP3*, *PeZFP36*, *PeZFP57*, *PeZFP66*), three members (*PeZFP3*, *PeZFP4*, *PeZFP57*), and two members (*PeZFP3*, *PeZFP57*) were highly expressed in roots, stems, and leaves, respectively (**Supplementary Table S4**). Generally, *PeZFP3* and *PeZFP57* were constitutively highly expressed across all three tissues (**Figure 7A**). Both genes contain the conserved QALGGH motif, and their promoter regions are enriched with various hormone-responsive and basal regulatory elements, suggesting their potential roles in fundamental physiological processes. Under salt stress, about half of the genes exhibited a trend of downregulation across tissues (**Figures 7B–D**). Five genes (*PeZFP4*, *PeZFP9*, *PeZFP33*, *PeZFP55* and *PeZFP57*) were markedly down-regulated, whereas four genes (*PeZFP15*, *PeZFP22*, *PeZFP38* and *PeZFP50*) were up-regulated.

Further analysis linking expression patterns with phylogenetic subfamilies and CRE compositions revealed significant correlations. Genes in subfamily IV generally showed low expression under normal conditions. However, under salt stress, certain genes, such as *PeZFP38*, exhibited significant and sustained upregulation across all three tissues. The expression induction ranged from 4.3- to 10.2-fold in roots, 6- to 10.4-fold in stems, and 28- to 63.7-fold in leaves. This gene aligns with the strong enrichment of stress-responsive elements such as ARE and MBS in the promoters of this subfamily, indicating that subfamily IV may play a central role in salt stress signaling. In contrast, some highly expressed Q-type genes under normal conditions, such as *PeZFP57*, was significantly downregulated after salt treatment. Their promoters contain both developmental-related elements and a limited number of stress-responsive elements, implying that their functions may be more biased toward growth regulation.

Salt stress response analysis showed that different genes exhibited distinct temporal and spatial expression patterns, including rapid, sustained, or delayed responses. For example, *PeZFP22* and *PeZFP38* were rapidly induced and maintained high expression across roots, stems, and leaves, representing core stress-responsive genes. In contrast, genes such as *PeZFP61* were induced only in specific tissues or at certain time points, reflecting regulatory complexity. These differential expression trends are likely determined by the type and combination of CREs in their promoters. For instance, rapidly induced genes often harbor dense clusters of stress-related elements such as ARE and ABRE. Collectively, *PeZFPs* exhibit distinct transcriptional regulatory patterns across tissues. Most *PeZFP* members were induced by salt stress, implying their potential involvement in the salt stress responses.

### 3.7 Subcellular localization of PeZFP38

To investigate the response of *PeZFPs* to abiotic stress, particularly salt stress, we selected the gene *PeZFP38*, which is significantly induced by salt stress based on transcriptome results, for study. Previously, subcellular localization of PeZFPs was predicted using TBtools, which indicated that all PeZFPs had the highest scores for nuclear localization. Specifically, PeZFP38 scored 14 for nuclear localization, with no notable scores for other compartments. Based on this prediction, the recombinant vector pBI121-*PeZFP38*-GFP was constructed, with vector pBI121-GFP serving as a control.

**Figure 8:**
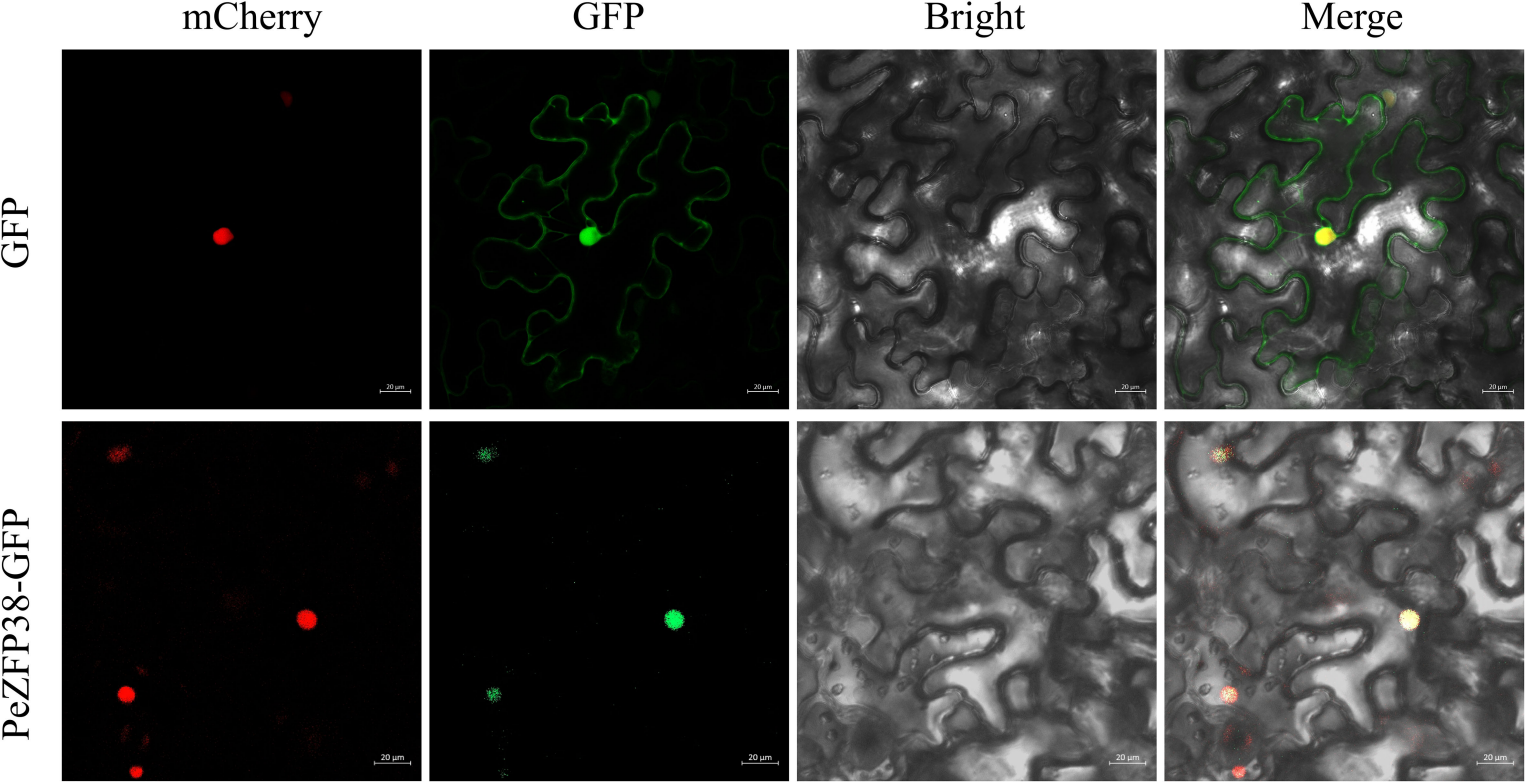
Subcellular localization of PeZFP38 in *N. benthamiana* epidermal cells. pBI121-GFP indicates the control. The confocal images of mCherry fluorescence (red), GFP fluorescence (green), bright-field, and merged-field. Bar = 20 μm.

Both constructs were co-transformed into *Agrobacterium* GV3101 together with the nuclear marker vector p2300-35S-D53-mCherry. After adjusting the bacterial suspension to an OD_600_ of 1.0, we performed transient co-expression in *N. benthamiana* leaves by infiltration. The leaves were incubated in darkness for 12 h, followed by 36 h under normal light conditions, after which fluorescence signals were observed using a laser confocal microscope. The results showed that the PeZFP38-GFP and mCherry signals completely overlapped within the nucleus, whereas the GFP signal in the control was detected throughout the entire cell (**Figure 8**). Therefore, PeZFP38 is a nuclear-localized protein, providing subcellular evidence for its function as a TF involved in the salt stress response, supporting a molecular mechanism whereby it mediates plant salt adaptation through transcriptional regulation within the nucleus.

### 3.8 Transient overexpression of *PeZFP38* enhanced the salt tolerance of poplar leaves

**Figure 9:**
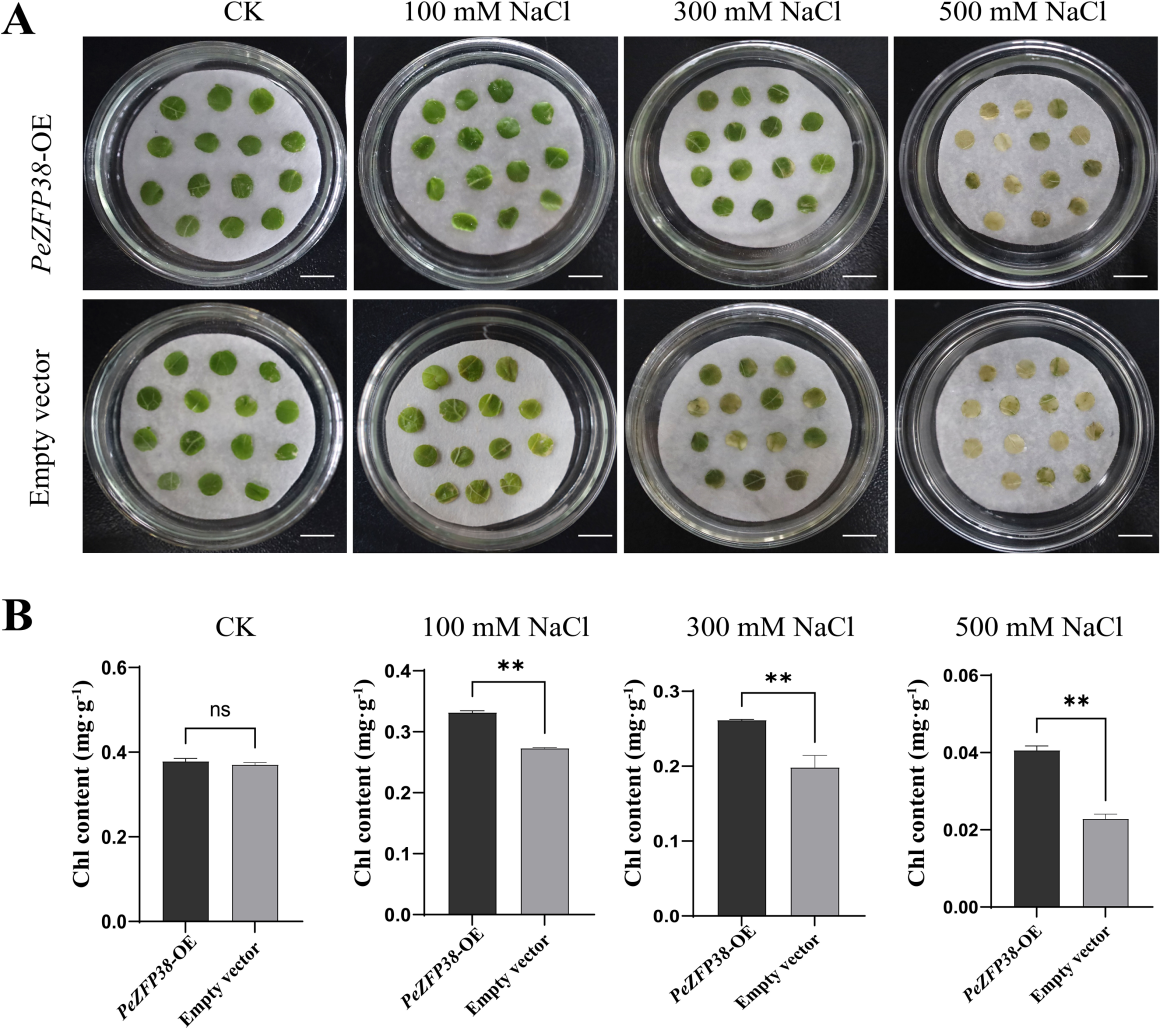
Transient overexpression of *PeZFP38* enhances salt tolerance in poplar leaves. **(A)** Phenotypes of poplar leaves transiently transformed with *PeZFP38*-OE and empty vector after 7 d of NaCl treatment. **(B)** Total chlorophyll content of leaves with *PeZFP38*-OE and empty vector after 7 d of NaCl treatment. Statistical significance was assessed employing the statistical T-test (**p < 0.01). Bar = 1 cm.

To investigate the function of *PeZFP38*, we conducted transient overexpression assays in leaves of the fast-growing poplar variety 84K, as the transient transformation system for *P. euphratica* itself has not yet been established. This approach preliminarily assessed the role of *PeZFP38* in salt tolerance. Successful introduction of *PeZFP38* was confirmed by genomic DNA analysis, and its expression level was about 8-fold higher than in the empty-vector control (**Supplementary Figure S1**), verifying an effective transient transformation system. Under salt treatment, leaves exhibited progressively severe chlorosis and wilting with increasing NaCl concentration (**Figure 9A**). Compared with leaves transformed with the empty vector, those overexpressing *PeZFP38* showed significantly reduced chlorosis at the same salt concentrations, indicating that *PeZFP38* may enhance the tolerance of plant to salt stress.

To further quantify these phenotypic differences, total chlorophyll content was measured (**Figure 9B**). Under 100 mM NaCl treatment, chlorophyll content in *PeZFP38*-overexpression (OE) leaves was approximately 1.2-fold higher than those in control. Under 300 mM NaCl treatment, it remained significantly higher (about 1.3-fold of the control). Even under 500 mM NaCl stress, chlorophyll in the *PeZFP38*-OE group was still 1.8-fold higher than in controls. Although chlorophyll declined with rising salt concentration in all treatments, overexpression of *PeZFP38* consistently alleviated chlorophyll degradation. These results indicate that *PeZFP38* helps maintain chlorophyll stability and positively regulates salt tolerance in poplar.

### 3.9 Overexpression of *PeZFP38* improved salt tolerance in *A. thaliana*

To further verify the role of *PeZFP38* in plant stress resistance, the overexpression vector pBI121-*PeZFP38*-GFP was introduced into *A. thaliana* via *Agrobacterium*-mediated transformation. Positive transgenic lines were confirmed by PCR amplification of the target gene (**Supplementary Figure S2**) and by assessing expression levels using qRT-PCR (**Supplementary Figure S3**). Three lines (OE2, OE5, and OE6) showing higher relative expression levels were selected for subsequent functional analyses.

**Figure 10:**
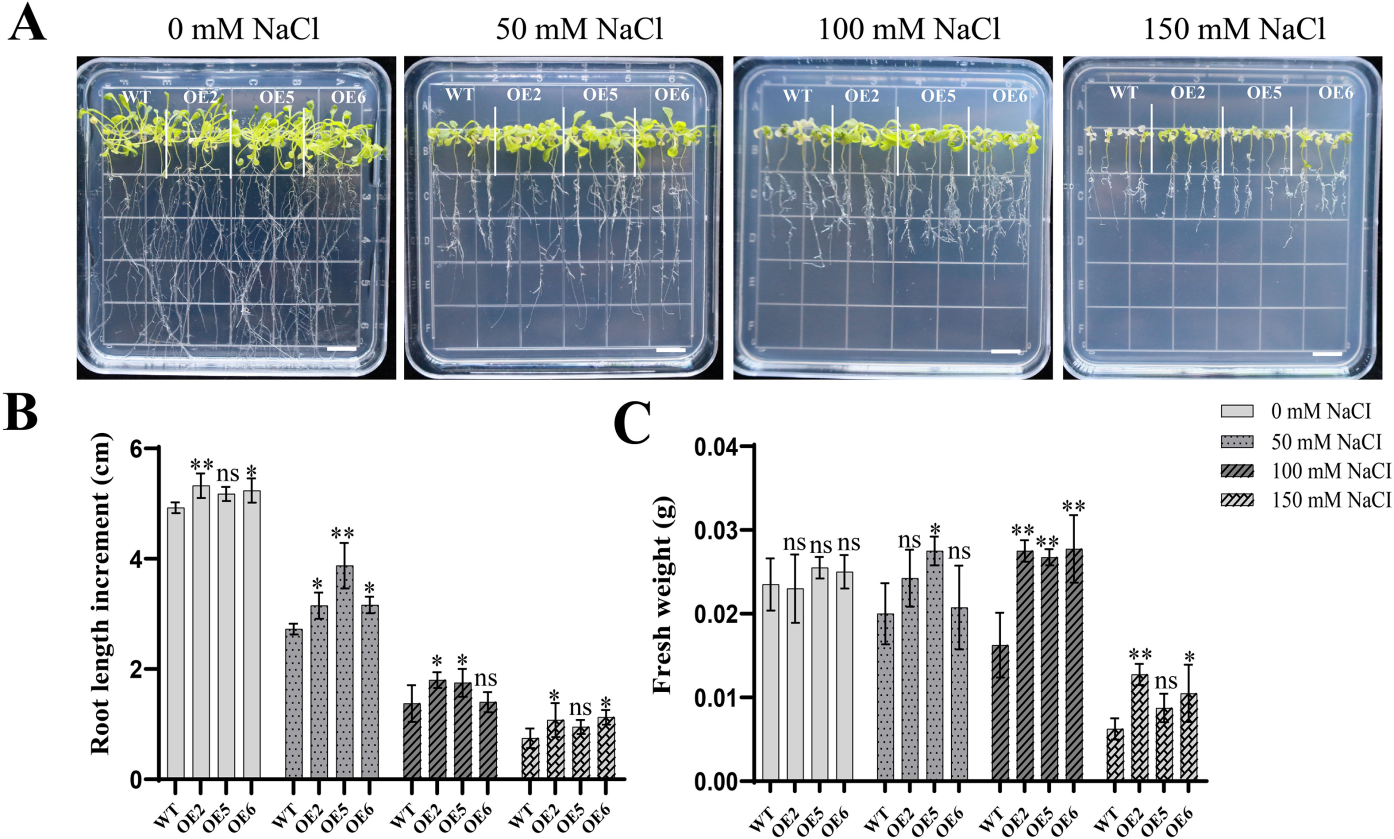
Overexpression of *PeZFP38* in *A. thaliana* increases root length increment and fresh weight under salt stress in plate culture. **(A)** Phenotypes of *PeZFP38*-OE plants and WT under normal conditions and after salt treatment. Bar = 1 cm. **(B)** Root length increment. **(C)** Fresh weight. Data represent the mean ± standard deviation (SD) from at least three independent biological replicates. Statistical significance was determined using one-way ANOVA (*p < 0.05; **p < 0.01). The same convention applies to the following figures.

Plate assays demonstrated that *PeZFP38* overexpression significantly improved salt tolerance. On medium containing 50, 100, or 150 mM NaCl, WT plants exhibited severe chlorosis and wilting, whereas the growth inhibition of *PeZFP38*-OE lines was markedly less pronounced (**Figure 10A**). Under 50 mM NaCl treatment, the root length increment of *PeZFP38*-OE lines was significantly greater than that of WT, with an average increase of approximately 24.6%. Under 100 mM NaCl treatment, the fresh weight of *PeZFP38*-OE lines showed a significant average increase of about 68.2% compared to WT (**Figures 10B, C**). Although the growth advantage of some *PeZFP38*-OE lines did not reach statistical significance across all salt concentrations, they consistently performed better than WT.

**Figure 11:**
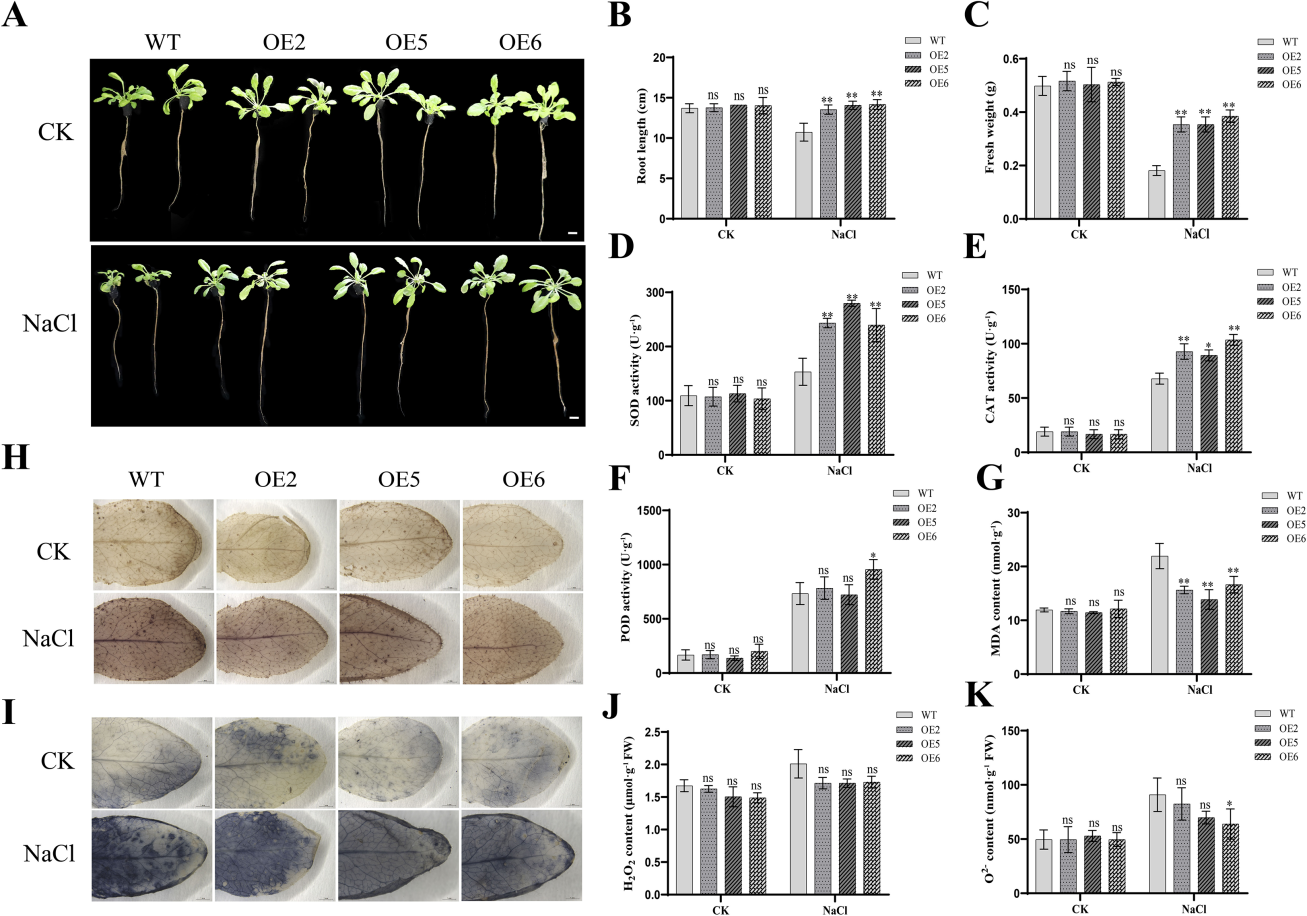
Overexpression of *PeZFP38* in *A. thaliana* enhances tolerance to salt stress and improves ROS scavenging ability under hydroponic conditions. **(A)** Phenotypes of *PeZFP38*-OE transgenic lines and WT under normal conditions and 100mM NaCl treatment for 7 d. Bar = 1 cm. **(B)** Root length. **(C)** Fresh weight. **(D)** SOD activity. **(E)** CAT activity. **(F)** POD activity. **(G)** MDA content. **(H)** DAB staining. **(I)** NBT staining. **(J)** H_2_O_2_ content. **(K)** O_2_^^-^^ content.

Hydroponic experiments further confirmed these findings. Under salt stress, the growth of *PeZFP38*-OE plants was significantly better than that of WT plants (**Figure 11A**), whereas WT plants growth was severely inhibited, showing yellowing and partial abscission. *PeZFP38*-OE lines showed a significantly smaller reduction in root length compared to WT, and their fresh weight accumulation averaged approximately 2.1 times that of WT (**Figures 11B, C**). Physiological analysis indicated enhanced antioxidant capacity in *PeZFP38*-OE plants. Under salt stress, the activities of superoxide dismutase (SOD) and catalase (CAT) in *PeZFP38*-OE leaves reached approximately 1.7-fold and 1.4-fold, respectively, of the levels in WT (**Figures 11D, E**). Peroxidase (POD) activity also showed an increasing trend (**Figure 11F**). Correspondingly, the malondialdehyde (MDA) content in *PeZFP38*-OE lines was only about 0.7 times that of WT, indicating significantly reduced membrane lipid peroxidation damage (**Figure 11G**). Histochemical staining provided visual support for these data. The color intensity of DAB and NBT staining serves as an indicator of the accumulation levels of hydrogen peroxide (H_2_O_2_) and superoxide anion (O_2_^^-^^), respectively. No obvious difference in staining intensity was observed among untreated leaves (**Figures 11H, I**). However, under salt stress conditions, *PeZFP38*-OE plants exhibited lower accumulation of H_2_O_2_ and O_2_^^-^^ compared to WT (**Figures 11J, K**). In summary, *PeZFP38* overexpression enhances salt tolerance in *Arabidopsis* by promoting biomass accumulation, strengthening the antioxidant enzyme system, and reducing the accumulation of ROS under salt stress.

Earlier analysis revealed that the promoter of *PeZFP38* was enriched with ABA-responsive elements (ABRE) and oxidative stress-associated elements (ARE). *PeZFP38* was persistently induced in roots, stems, and leaves of *P. euphratica* under salt stress. Moreover, heterologous expression of *PeZFP38* in *Arabidopsis* significantly enhanced the activities of antioxidant enzymes such as SOD and CAT. In summary, *PeZFP38* likely functions as a transcriptional regulator that enhances salt tolerance by integrating ABA signaling with ROS scavenging systems, thereby activating downstream antioxidant and stress-adaptive genes, which collectively boost antioxidant capacity and mitigate oxidative damage.

## 4 Discussion

Zinc finger proteins constitute one of the largest families of TFs in plants, with C2H2-type zinc finger TFs being the most extensively studied. They play pivotal roles not only in regulating transcription during plant growth and development but also in responding to environmental stresses (Xie et al., 2019). To date, research on C2H2-ZFP gene family has predominantly focused on model plants such as *A.thaliana* (Lu et al., 2024) and tomato (T. Zhao et al., 2020). However, a systematic investigation encompassing genome-wide identification, expression profiling, and functional validation in the highly salt-tolerant tree species *P. euphratica* remains lacking.

In this study, we identified 67 PeZFPs, all of which contain zinc finger domains. This finding is consistent with previous reports on the C2H2-ZFP gene family (Pu et al., 2021). The number of *PeZFPs* is lower than that in its close relative *P. trichocarpa* (109 members) (Liu et al., 2015). This discrepancy may stem from different identification thresholds, but it could also indicate lineage-specific contraction and functional refinement of the gene family post-speciation. Based on protein similarity and evolutionary relationships, we classified the PeZFPs into five subfamilies. Similar subfamily classification results from other species (Liu et al., 2024; Zhou et al., 2024) support the robustness of this evolutionary framework. Notably, the expansion of the PeZFP gene family was driven by both tandem and segmental duplications, coupled with strong purifying selection. This evolutionary pattern, characterized by stable genomic duplication followed by selection pressure, is typical for stress-related gene families adapting to long-term environmental challenges (Singh and Sharma, 2024). It likely represents a key molecular underpinning for the adaptation of *P. euphratica* to its harsh, saline-alkaline riparian habitat. Furthermore, the promoter regions of *PeZFPs* were widely enriched with stress-responsive CREs such as ARE, ABRE, and MBS, reinforcing the hypothesis of an adaptive evolutionary shift toward a stress response function in this family.

Within this evolutionary framework, members exhibited significant structural and potential functional divergence. The vast majority *PeZFPs* contained the plant-specific QALGGH motifs, which is conserved in species like cucumber (Yin et al., 2020), *Panax ginseng Meyer* (Jiang et al., 2022), and *Glycine soja* (Liu et al., 2020). A total of 54 Q-type (80.6%) C2H2-ZFPs may function as transcriptional regulators broadly involved in stress responses. However, this assumption requires further experimental verification. For instance, many plant Q-type C2H2-ZFPs utilize EAR motifs to repress target genes, balancing growth and defense under stress (Xie et al., 2019). For example, *PlZAT10* regulates the release of dormancy in peony seeds by binding to the promoter of the ABA catabolic gene *PlCYP707A2* (Song et al., 2024). Importantly, under salt stress, most *PeZFPs* showed tissue-specific induction or repression patterns. These expression dynamics are closely linked to the specific combinations of hormone-related (e.g., ABA, jasmonate) and stress-responsive elements in their promoters, forming the basis of a refined transcriptional regulatory network for salt stress adaptation in *P. euphratica*. For example, some genes (e.g.,*PeZFP3*, *PeZFP61*) specifically highly induced in roots, whose promoters may be enriched with hypoxia (ARE) or root development-related elements, could play a role in the early perception and relay of salt stress signals in roots.

The findings indicate that most *PeZFPs* participate in pathways related to abiotic stress responses. Among them, *PeZFP38* emerged as a key focus for functional analysis due to its multiple salient features. It belongs to subfamily IV, which contains *P. euphratica*-specific clades, hinting at possible functional innovation within this lineage. Its promoter is densely packed with hormone-related (e.g., ABRE for ABA response) and stress-responsive CREs. Under salt stress, *PeZFP38* was rapidly and strongly induced in roots, stems, and leaves (with fold-changes up to 4.3–63.7), highlighting its important role in regulating response of poplar to salt stress. Heterologous overexpression experiments demonstrated that *Arabidopsis* lines overexpressing *PeZFP38* significantly enhanced salt tolerance in, evidenced by increased biomass, elevated antioxidant enzyme (SOD, CAT) activities, and reduced oxidative damage (MDA). Based on these findings, we propose a mechanistic hypothesis for *PeZFP38*. Its role in enhancing antioxidant capacity suggests it may mitigate salt-induced oxidative damage by regulating key genes in the ROS scavenging pathway. A precedent exists in tree stress resistance. For instance, *PeSTZ1* in *P. euphratica* was shown to enhance ROS scavenging and freezing tolerance by directly regulating the peroxidase gene *PeAPX2* (He et al., 2020). In *Betula platyphylla*, *SZA1* enhances salt tolerance by improving ROS scavenging capacity and proline accumulation, and is involved in the ABA/JA signaling pathways (Zhang et al., 2022b). Therefore, PeZFP38 likely functions as a TF that integrates upstream stress signals (potentially mediated by ABRE elements linked to the ABA pathway) to activate the transcription of downstream antioxidant and stress-protective genes, ultimately enhancing stress tolerance by maintaining ROS homeostasis (Bokolia et al., 2024). This framework logically connects the CRE features in its promoter, its strong induction pattern, and the observed physiological function of enhanced antioxidative defense.

Despite systematically identifying the PeZFP gene family and preliminarily revealing the function of *PeZFP38*, this study has limitations that outline future research directions. Our functional validation relied on a heterologous overexpression system. To conclusively demonstrate the necessity of *PeZFP38* for salt tolerance in *P. euphratica*, subsequent studies should employ gene editing (e.g., CRISPR/Cas9-mediated knockout) or complementation tests in poplar. Future studies will utilize molecular biology techniques, such as ChIP-seq, to directly identify its downstream target genes, thereby mapping the complete antioxidant or ion homeostasis pathways it regulates. Although we predicted numerous CREs, the specific elements critical for driving the salt-induced expression of *PeZFP38* require validation through promoter deletion or mutation analyses. This study did not investigate core salt tolerance parameters in *P. euphratica*, such as K^+^/Na^+^ homeostasis. Linking the function of *PeZFP38* to these core ion homeostasis indicators would significantly enhance the physiological depth of the research.

## 5 Summary

In this study, we identified 67 C2H2-ZFP proteins in *P. euphratic*a for the first time and classified them into five subfamilies, all containing zinc finger domains. Further evolutionary analysis indicated adaptive expansion of the family via duplication and purifying selection, establishing a preliminary framework for understanding functional divergence among its members. Phylogenetic analysis revealed that *P. euphratica* shares a closer evolutionary relationship with *P. trichocarpa* than with *A. thaliana*. Transcriptome analysis showed that most *PeZFPs* respond to salt stress and exhibit tissue-specific expression patterns, with *PeZFP38* being significantly induced by salt stress. Both transient expression of *PeZFP38* in poplar leaves and its stable overexpression in *A. thaliana* enhanced plant salt tolerance, which likely exerts its function by activating the ROS scavenging pathway. Collectively, this work represents the first systematic characterization of the C2H2-ZFP family in *P. euphratica* and demonstrates the key role of *PeZFP38* in salt stress response. As a valuable candidate gene, *PeZFP38* provides important genetic resources and a theoretical basis for elucidating the molecular mechanisms of salt tolerance in trees and promoting stress-resistant molecular breeding.

## Data Availability

The datasets presented in this study can be found in online repositories. The names of the repository/repositories and accession number(s) can be found in the article/**Supplementary Material**.

## Supplementary material

Supplementary Table 1 The physicochemical characteristics of PeZFP proteins in *P. euphratica*.

Supplementary Table 2 The type and motif sequences of PeZFP proteins in *P. euphratica*.

Supplementary Table 3 Ka/Ks values of *PeZFPs* duplicated gene pairs in *P. euphratica*.

Supplementary Table 4 The expression patterns of *PeZFPs* in various tissues and under salt stress.

Supplementary Table 5 Primers used in this study.

Supplementary Table 6 List of Abbreviations for Proper Nouns.

Supplementary Figure 1 qRT–PCR for *PeZFP38* expression in *PeZFP38*-overexpressing leaves in *Populus*.

Supplementary Figure 2 PCR detection of *Arabidopsis* lines overexpressing *PeZFP38*.

Supplementary Figure 3 qRT–PCR for *Arabidopsis* lines overexpressing *PeZFP38*.
