# Supplementary material for: Genome-wide identification of the C2H2 zinc finger gene family in Populus euphratica and the functional analysis of PeZFP38 under salt stress
Source: Front Plant Sci. 2026 Jan 26;17:1754976. doi: 10.3389/fpls.2026.1754976 (PMC12885089; doi:10.3389/fpls.2026.1754976)
Supplement: Supplementary Figure 1 — qRT–PCR for PeZFP38 expression in PeZFP38-overexpressing leaves in Populus. [file DataSheet1.docx]

Supplementary Figures

**
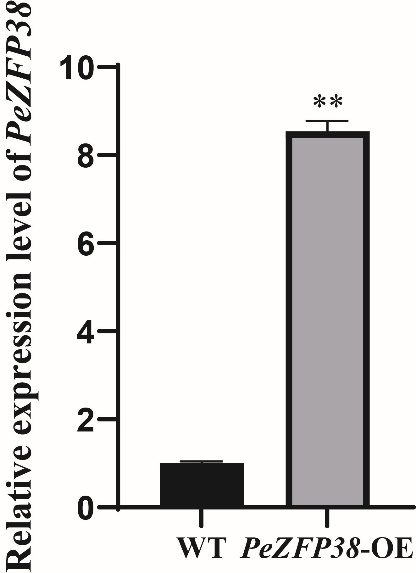
**

**Figure. S1** qRT–PCR for *PeZFP38* expression in *PeZFP38*-overexpressing leaves in *Populus* (**, *p* < 0.01).


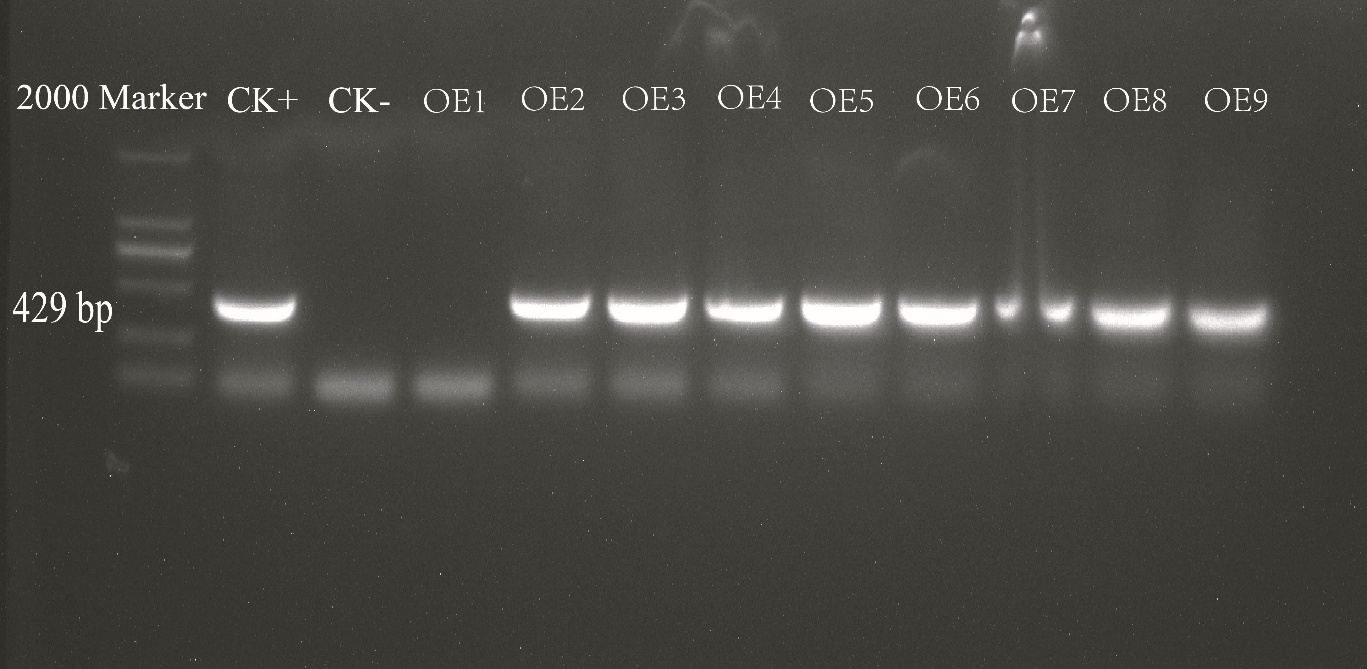


**Figure. S2** PCR detection of *Arabidopsis* lines overexpressing *PeZFP38*.


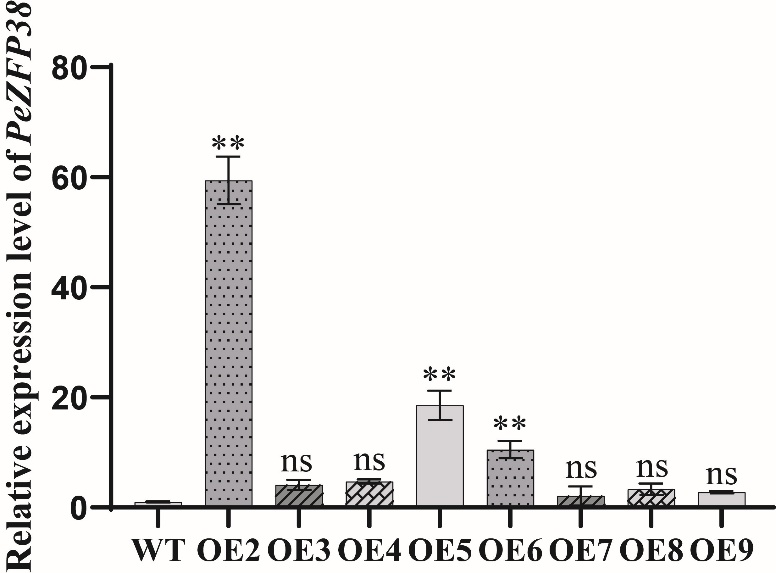


**Figure. S3** qRT–PCR for *Arabidopsis* lines overexpressing *PeZFP38* **(****, *p* < 0.01**).**
